# System Implementation Trade-Offs for Low-Speed Rotational Variable Reluctance Energy Harvesters

**DOI:** 10.3390/s21186317

**Published:** 2021-09-21

**Authors:** Ye Xu, Sebastian Bader, Michele Magno, Philipp Mayer, Bengt Oelmann

**Affiliations:** 1 Department of Electronics Design, Mid Sweden University, 85170 Sundsvall, Sweden; ye.xu@miun.se (Y.X.); michele.magno@pbl.ee.ethz.ch (M.M.); bengt.oelmann@miun.se (B.O.); 2 Department of Information Technology and Electrical Engineering, ETH Zurich, 8092 Zurich, Switzerland; mayerph@iis.ee.ethz.ch

**Keywords:** energy harvesting, rotational energy harvesting, kinetic energy harvesting, variable reluctance, self-powered sensors, internet of things, smart sensors

## Abstract

Low-power energy harvesting has been demonstrated as a feasible alternative for the power supply of next-generation smart sensors and IoT end devices. In many cases, the output of kinetic energy harvesters is an alternating current (AC) requiring rectification in order to supply the electronic load. The rectifier design and selection can have a considerable influence on the energy harvesting system performance in terms of extracted output power and conversion losses. This paper presents a quantitative comparison of three passive rectifiers in a low-power, low-voltage electromagnetic energy harvesting sub-system, namely the full-wave bridge rectifier (FWR), the voltage doubler (VD), and the negative voltage converter rectifier (NVC). Based on a variable reluctance energy harvesting system, we investigate each of the rectifiers with respect to their performance and their effect on the overall energy extraction. We conduct experiments under the conditions of a low-speed rotational energy harvesting application with rotational speeds of 5 rpm to 20 rpm, and verify the experiments in an end-to-end energy harvesting evaluation. Two performance metrics—power conversion efficiency (PCE) and power extraction efficiency (PEE)—are obtained from the measurements to evaluate the performance of the system implementation adopting each of the rectifiers. The results show that the FWR with PEEs of 20% at 5 rpm to 40% at 20 rpm has a low performance in comparison to the VD (40–60%) and NVC (20–70%) rectifiers. The VD-based interface circuit demonstrates the best performance under low rotational speeds, whereas the NVC outperforms the VD at higher speeds (>18 rpm). Finally, the end-to-end system evaluation is conducted with a self-powered rpm sensing system, which demonstrates an improved performance with the VD rectifier implementation reaching the system’s maximum sampling rate (40 Hz) at a rotational speed of approximately 15.5 rpm.

## 1. Introduction

Energy harvesting has drawn much interest due to its capability of extending battery lifetime, or even enabling fully energy-autonomous operation of new generations of smart sensors and internet of things edge devices [1,2,3]. It can be employed in a number of different application domains, ranging from biomedical engineering, via industrial applications, to structural health monitoring [4,5,6]. A typical energy harvesting system includes an energy transducer, an interface circuit, and a load that uses the accumulated energy to perform a specific task [3,7,8,9]. The energy transducer converts energy from the physical domain to the electrical domain, generating a source-dependent output signal [10,11]. For kinetic energy harvesting, electromagnetic and piezoelectric energy transducers are common choices, which create an alternating current (AC) output [1,7,12,13,14]. The intended system loads are commonly low-power electronic systems, such as Internet of Things (IoT) nodes or sensing systems, requiring a direct current (DC) input [9]. In order to effectively supply power to these loads, the interface circuit needs to perform AC-DC conversion, impedance matching, voltage regulation, and potentially energy buffering. A typical implementation combines a rectifier circuit with a power management integrated circuit (PMIC) [12,15,16,17,18].

Variable reluctance energy harvesting (VREH) has been identified to be a suitable technique for rotational kinetic energy harvesting with low rotational speeds [19,20,21,22,23]. VREHs are a type of electromagnetic energy harvesters, which generate an AC output as the result of magnetic flux changes inside a pickup coil [11,20]. As opposed to many other electromagnetic transducers, VREHs do not require a relative translation between coil and magnet, which makes them suitable for applications with large diameter rotating objects, as well as low rotational speeds [20,21]. However, the output voltage and frequency of VREH transducers is low at low rotational speeds [21,23], making the system design susceptible to interface circuit implementation. In [22], a self-powered rpm sensing system based on an VREH implementation was proposed. The system uses a Cockroft-Walton voltage multiplier in combination with a commercial LTC3588-1 PMIC as interface circuit. In the system analysis, it was shown that the power delivered to the load was below 20% of the power that can be obtained with a matched impedance load. Consequently, significant improvements on the system’s interface circuit are possible in order to optimize the electrical circuit for the power conversion.

Other studies have investigated and compared interface circuit designs for energy harvesting systems [3,12,16,24,25,26,27,28,29,30], as well as self-powered sensor systems incorporating a number of these interface circuits [17,18,31,32,33,34]. Many of these studies are generic without specific application constraints [3,12,29], whereas others are focused on piezoelectric transducers with relative high output voltages [15,18,27,30,35,36]. Interface circuits for electromagnetic transducers with relative low voltages have been addressed in [17,28,33,34,37]. In [28], Ulusan et al. proposed and evaluated an integrated interface circuit, including AC-DC and DC-DC conversion, based on an active voltage doubler (VD). However, active rectifier solutions may create cold-start challenges and are more difficult to be implemented effectively with discrete components, which is desirable for small-scale applications. Discrete interface circuit solutions have been implemented based on a passive VD and a discrete PMIC in [17,33], and based on a negative voltage converter rectifier (NVC) and PMIC in [34]. The difference in transducers being used in these studies makes a direct comparison between the respective interface circuit implementation difficult. Moreover, none of the studies related the converted power to that of a matched impedance load, which has been argued to be an important metric for energy harvesting interface circuits [24,27,30,32,35,36,38,39].

**Figure 1:**
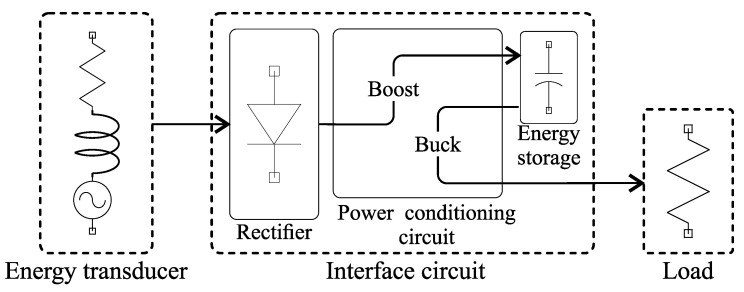
Overview of the Variable Reluctance Energy Harvesting system under evaluation.

In this paper, we conduct a systematic comparison of interface circuits based on three passive rectifier implementations for a VREH system as depicted in Figure 1. The rectifiers under evaluation are a full-wave bridge rectifier (FWR) [18,29,40,41,42], a Delon full-wave voltage doubler (VD) [33,40,43,44,45], and a negative voltage converter (NVC) [29,34,42,46,47]. System implementations using these rectifiers in combination with a state-of-the-art DC-DC PMIC (TI BQ25570) are evaluated experimentally under the conditions of a low-speed rotational energy harvesting application. In order to evaluate the interface circuits, power conversion efficiency (PCE) and power extraction efficiency (PEE) are used as metrics. While PCE is limited to the losses within the interface circuits [48], PEE includes effects on the transducer by relating the obtained output power to the output power with a matched impedance load [24,30,32,38]. Moreover, the achievable sampling rates of a self-powered rpm sensing system based on the respective interface circuits are investigated.

The key contributions of this study are summarized as follows: (i) a systematical and experimental evaluation of interface circuits based on different rectifiers, including their losses and effects on the extracted power. This particularly enables a direct comparison between VD and NVC; (ii) a demonstration that the optimum rectifier selection can change over the range of operating conditions in a VREH system implementation; (iii) a self-powered, wireless RPM sensing system operating at low rotational speeds with an PEE of up to 70%.

## 2. Variable Reluctance Energy Harvesting System

A high-level architectural overview of the targeted variable reluctance energy harvesting system is given in Figure 1. It can be divided into three core modules, namely the variable reluctance energy transducer, the interface circuit including AC-DC and DC-DC conversion, as well as the electrical load.

### 2.1. Variable Reluctance Energy Harvester

The variable reluctance energy harvester is a type of electromagnetic energy harvester, which operates based on Faraday’s law of induction. A VREH generates an electromotive force due to changes in magnetic flux, and it can for example be used in rotational kinetic energy harvesting applications [21]. In a VREH, both the magnet and the pickup coil are stationary, whereas the reluctance variation, and thus the changes in magnetic flux, are a result of the relative motion between a ferromagnetic structure and the pickup unit.

**Figure 2:**
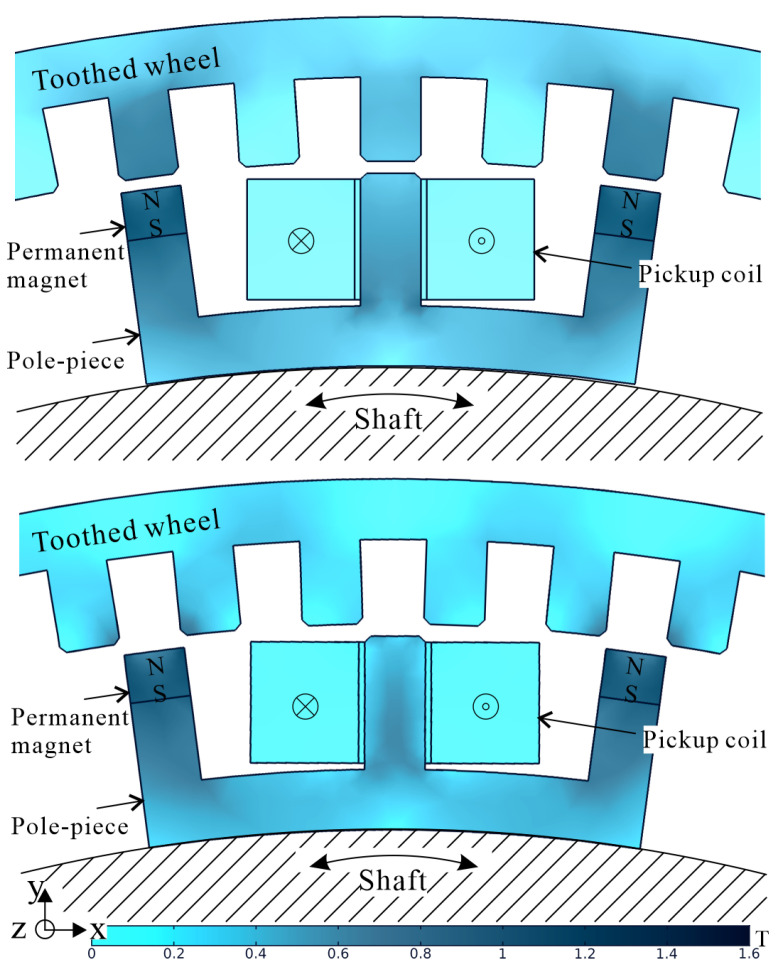
Magnetic flux density distribution of the VREH at the two extreme positions aligned position (**top**) and unaligned position (**bottom**).

In this work, an m-shaped, on-rotor VREH structure is used [22]. This structure is illustrated in Figure 2. The magnetic flux distribution varies periodically between the two extreme positions in the core of the pickup coil as the shaft rotates. As a result, an alternating voltage is induced across the pickup coil, with a frequency of

(1) $$f_{c}=\omega N_{t}\;,$$

where ω is the rotational speed in rad/s, and $N_{t}$ is the number of teeth of the tooth wheel.

To maximize energy extraction from the VREH, a complex conjugate matched impedance is required as load to the pickup coil. As the VREH transducer is inductive, its complex conjugate matched impedance can be implemented with a resistor $R_{\mathrm{Load}}$ and a capacitor $C_{\mathrm{Load}}$, such that

(2) $$R_{\mathrm{Load}}=R_{\mathrm{Coil}}$$

(3) $$C_{\mathrm{Load}}=\frac{1}{{\left(2\pi f_{c}\right)}^{2}\;\cdot \;L_{\mathrm{Coil}}}\;,$$

where $R_{\mathrm{Coil}}$ and $L_{\mathrm{Coil}}$ are the DC resistance (DCR) and internal inductance of the pickup coil. By means of the maximum power transfer theorem, connecting an impedance matched load to the pickup coil can determine the maximum amount of power that is dissipated in the load, presenting the power extraction ability of the VREH transducer. This is used to derive one of the key performance metrics, power extraction efficiency, further discussed in Section 3.3. The maximum amount of power is a function of multiple system parameters and operating conditions and can be estimated as [21]

(4) $$P_{\max}=\frac{N_{\mathrm{Coil}}^{2}}{8R_{\mathrm{Coil}}}\Delta_{\phi }^{2}{\left[\frac{\omega N_{\mathrm{tooth}}}{2\pi }\right]}^{2}\;.$$

where $N_{\mathrm{Coil}}$ is the number of coil turns, $\Delta_{\phi }$ is the magnetic flux difference between aligned and unaligned positions shown in Figure 2, ω is the angular velocity, and $N_{\mathrm{tooth}}$ refers to the number of teeth in the toothed wheel. Neglecting the inductive part of the coil’s impedance, an accurate estimation is only achieved at low operation frequencies of $f_{c}$.

### 2.2. Interface Circuit

The interface circuit is composed of two units, a rectifier circuit (AC-DC) and a power conditioning circuit (DC-DC). For the power conditioning circuit, an off-the-shelf DC-DC PMIC of type Texas Instruments BQ25570 is used. This PMIC is an inductive switching regulator that boosts the input voltage to charge an intermediate storage device (e.g., a supercapacitor or battery). The energy that is stored is then made available to the load via an additional integrated buck converter.

**Figure 3:**
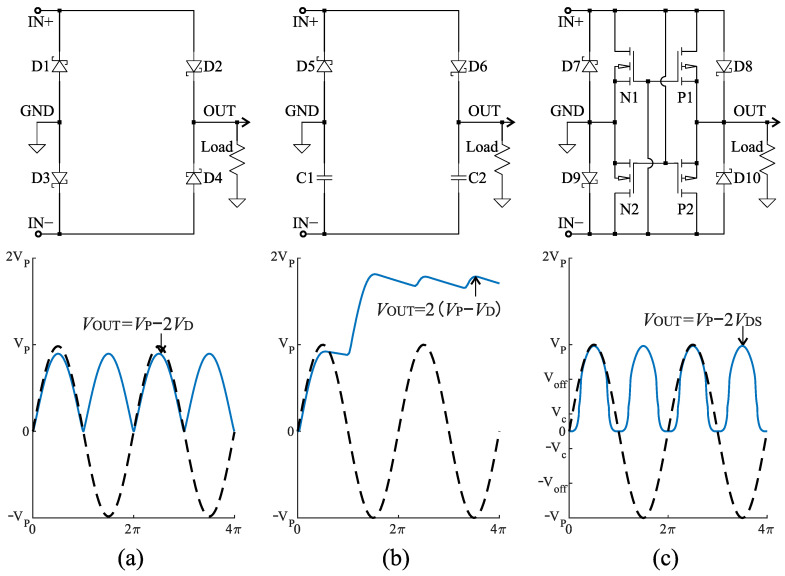
Schematic diagrams of the three rectification circuits, (**a**) full-wave diode bridge rectifier (FWR), (**b**) Delon full-wave voltage doubler (VD), and (**c**) negative voltage converter (NVC), and associated qualitative waveforms of input and output voltage. $V_{P}$ is the amplitude of the input voltage across *IN+* and *IN−*.

For the rectifier circuit, three implementations commonly used in low-power energy harvesting systems have been chosen, which are evaluated separately and compared to each other. The three rectifier implementations are a full-wave diode bridge rectifier (FWR), a Delon full-wave voltage doubler (VD), and a negative voltage converter (NVC). Circuit implementations for these three rectifiers are depicted in Figure 3, together with their ideal input and output voltages. The terminals *IN+* and *IN−* of a rectifier are connected to the pickup coil to maintain the differential input. A rectified voltage is produced at the terminal *OUT* with a reference voltage on the terminal *GND*.

The FWR consists of four diodes arranged in the form of a bridge. This circuit topology is the most common circuit used in energy harvesting applications due to its simple operation and implementation. During the positive half-cycle of the input voltage across *IN+* and *IN−*, *D2* and *D3* are forward-biased and conduct current. Conversely, during the negative half-cycle of the input voltage, *D1* and *D4* are forward-biased and conduct current, but *D2* and *D3* are reverse-biased. As a result, a full-wave rectified voltage is produced at its output. However, the output voltage level is reduced by the voltage drops of the two diodes. In most cases, a filter solution, such as a smoothing capacitor, is connected in parallel with the load across the output terminals reducing the ripple of the output DC voltage.

The VD (or single stage voltage multiplier) consists of diodes and capacitors to rectify and boost the input voltage across *IN+* and *IN−*. Its output voltage is twice that of the input AC peak-voltage $V_{P}$. During the positive half cycle of the input, the diode *D6* is forward biased, and the current flows though the diode and charges the capacitor *C2* to a voltage of $V_{P}$. Similarly, during the negative cycle *C1* is charged through *D5* to $V_{P}$. The output voltage across both the capacitor thus is $2V_{P}$. For a real implementation, the charge on each capacitor is reduced by the forward voltage drop of the diodes ($V_{D}$).

Based on a full gate cross-coupled topology, an NVC uses MOSFETs instead of diodes, and converts the negative half of the input voltage across *IN+* and *IN−* to positive ones for voltage rectification. Due to the low on-resistance of the MOSFETs, the NVC typically incurs a lower forward voltage drop ($V_{\mathrm{DS}}$), defined by the the sum of drain-source voltages of two conducting MOSFETs. As shown in Figure 3c, during the positive half-cycle, *P1* and *N2* are turned on, and *N1* and *P2* are turned off. The current flows from *IN+* to *OUT* via *P1* and returns to *IN−* over *N2*. Therefore, the forward voltage drop is defined by *P1* and *N2* during the positive half-cycle. Moreover, the influence of the on-resistance causes an increase in $V_{\mathrm{OUT}}$ when the input voltage $V_{p}$ surpasses a critical value $V_{c}$ [49]. When $V_{p}$ reaches $V_{\mathrm{off}}$, the NVC operates correctly and provides a low on-resistance conduction path for current to flow, where $V_{\mathrm{off}}$ depends on the highest threshold voltage $V_{\mathrm{GS}(\mathrm{th})}$ between *N*- and *P*-MOSFETs. In this study, Schottky diodes are used in parallel with each MOSFET to adapt to a low-voltage rectification, because the forward voltage of the Schottky diodes is lower than $V_{\mathrm{GS}(\mathrm{th})}$ of the MOSFETs [34,47].

**Figure 4:**
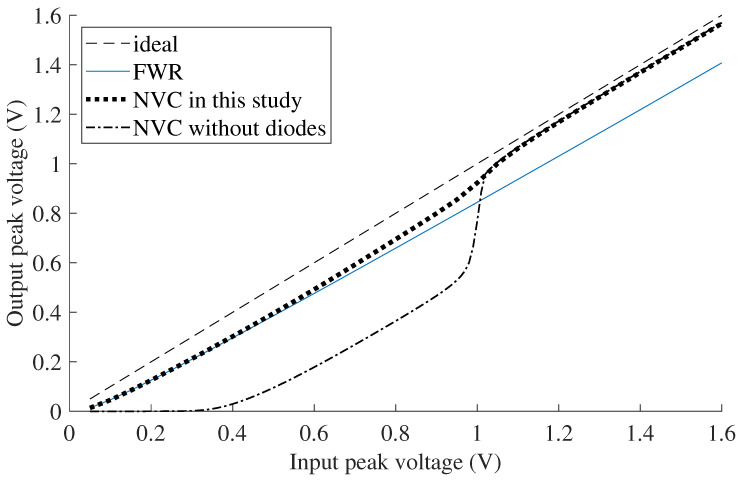
Simulated input and output voltage of the rectifiers using the circuits shown in Figure 3, with an AC voltage source of 7.55 Hz and a load of 10 kΩ.

The effect of this can be observed in Figure 4. With input voltages greater than $V_{\mathrm{off}}$ (approximately 1.2 V), the input voltage is approximately equal to the output voltage, resulting in a high conversion efficiency. With help of the parallel connected Schottky diodes, the NVC acts as an equivalent FWR at low input voltages of 0.1 V to 0.8 V. In contrast, the NVC without the additional diodes has significantly lower rectification performance, and is practically unusable at voltages below 0.4 V.

### 2.3. Wireless RPM Sensing System

**Figure 5:**
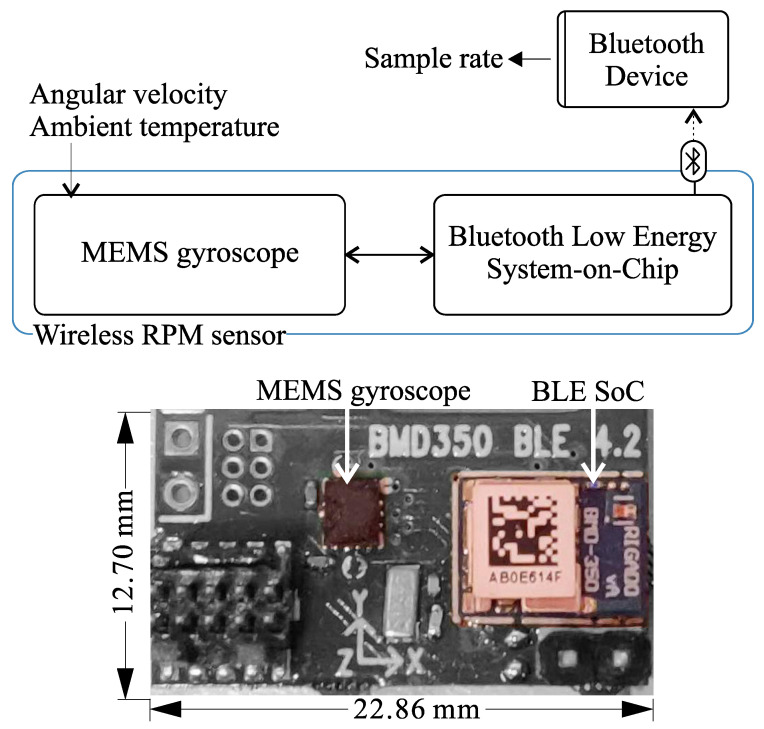
Block diagram of the wireless RPM sensing system (**top**), and the sensor node implementation (**bottom**), including a Bosch BMG250 MEMS gyroscope and a Rigado BMD350 module based on a Nordic Semiconductor nRF52832 SoC.

The intended electrical load in this study is a wireless RPM sensing system, as illustrated in Figure 5. The system’s main components are a MEMS gyroscope and a Bluetooth Low Energy (BLE) System-on-Chip (SoC). The rotational speed of the host structure is thus obtained by measuring the angular velocity using the gyroscope. The BLE SoC transmits the measurements, together with ambient temperature readings, to a Bluetooth host device, where the data will be further processed and used. The MEMS gyroscope is a Bosch BMG-250 with low current draws of 3 μA and 850 μA at 1.8 V in idle and data acquisition mode, respectively.

**Figure 6:**
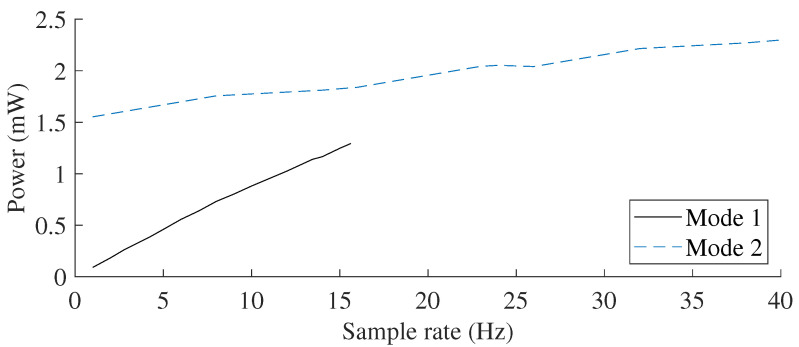
The average power requirement as a function of sample rate for the wireless sensor working in the two operating modes with a constant supply voltage of 1.8 V.

**Figure 7:**
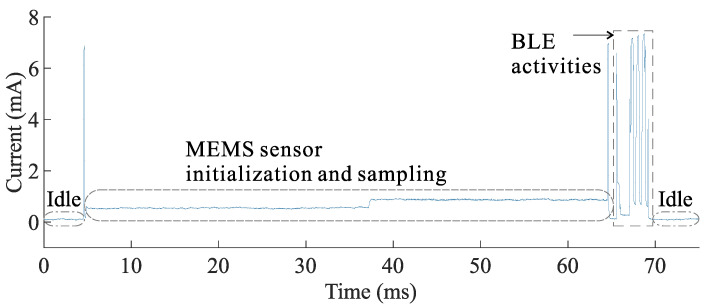
The current profile for the wireless sensor working in operating mode 1. The sensor is operated with a constant supply voltage of 1.8 V.

The sensing system can be operated in different operating modes, based on the duty-cycling of its sub-systems. These operating modes affect the system’s achievable sample rate and its average power consumption, as shown in Table 1 and Figure 6. In mode 1, both the sensing and communication sub-systems are duty-cycled. This results in the lowest power consumption when the system is idle with a current draw of 5 μA, but requires the MEMS sensor (BMG-250) to be reinitialized for every sample. Due to the relatively long initialization time of the gyroscope (59 ms), the maximum achievable sample rate in this mode of operation is limited to 15.625 Hz and the contribution to the total energy consumption is high ( 63.67 μJ). In order to further increase the sample rate, in mode 2, the MEMS gyroscope remains always on avoiding initialization, whereas the BLE continues to be duty-cycled. The sample rate in this mode is limited to a maximum of 40 Hz due to the minimum advertisement interval of the BLE unit of 25 ms. An example of the current consumption profile for operating mode 1 is depicted in Figure 7.

The targeted applications of the sensing system are angular speed measurements for rotating objects with low-speed and large shaft diameters. The VREH is a suitable technique for such applications, making the sensing system energy autonomous. Specific constraints of a machine health monitoring system for a hydraulic motor [50] have been considered, leading to an investigation of the proposed system in a laboratory test bench with rotational speeds of 5 rpm to 20 rpm and a shaft diameter of 270 mm. More details on the experimental setup are presented in Section 3.

## 3. Experimental Setup

**Figure 8:**
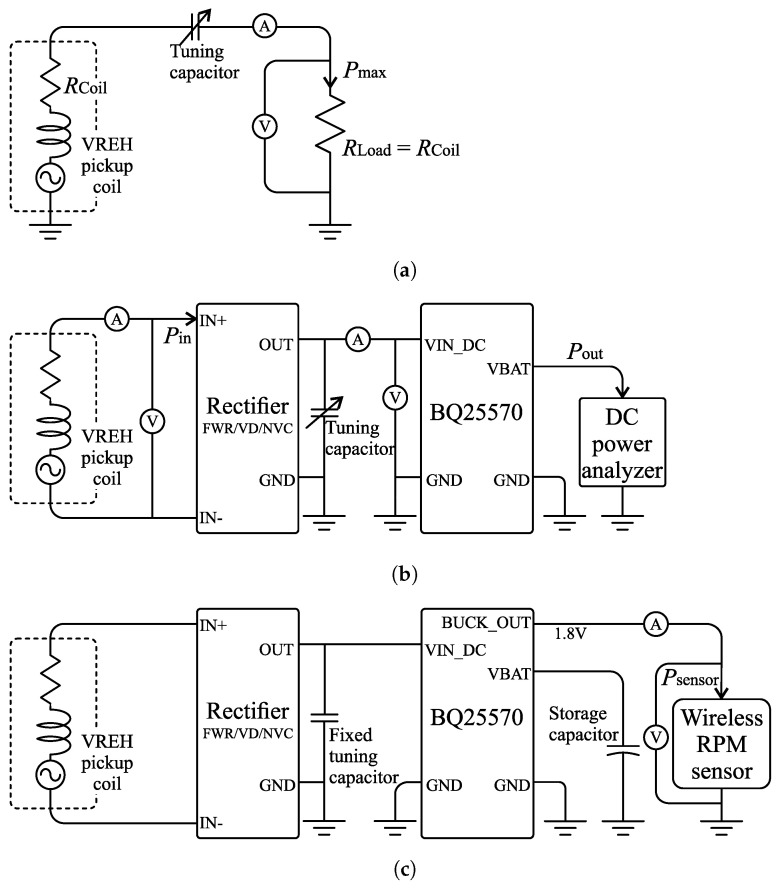
Schematic diagrams of the experimental setups for (**a**) evaluating maximum output power under impedance matched load; (**b**) evaluation of the interface circuit implementations; and (**c**) the full system evaluation with a wireless RPM sensor as the electrical load

To evaluate the effect of the rectifier selection on the system performance, three sets of experiments are conducted. In the first set of experiments, the maximum output power of the VREH transducer is determined. For this, the output power is acquired under different loads for given excitation conditions. In the second set of experiments, the circuit losses and the DC output power of the system are investigated. This allows to obtain general performance metrics of the individual implementations, and compare them to each other. In the third set of experiments, the wireless RPM sensor is attached to the VREH as a load. These experiments provide information on the effects of the gained performance improvements on the final system operation. Schematic overviews of the experimental setup for each set of experiments are provided in Figure 8, and more details are provided in Section 3.2.

For the interface circuit implementation, a printed circuit board (PCB) containing the three rectifier circuits has been produced. Component selection has a significant influence on the rectifier performance, and care has been taken to select diodes and MOSFETs with beneficial properties (e.g., low forward voltage drop). Table 2 provides a list of the selected components, as well as their characteristics. A tuning capacitor is provided at the output of the rectifier circuit. In order to simplify the evaluation of different capacitance values, it is provided in form of a capacitor decade box with capacitance ranges of 1 μF to 4000 μF.

### 3.1. Mechanical Setup and Test Conditions

**Figure 9:**
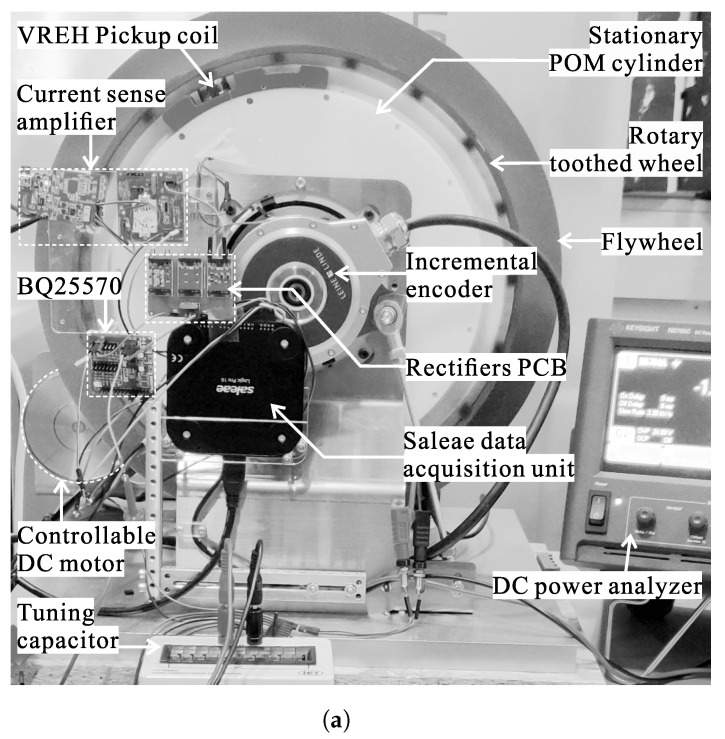

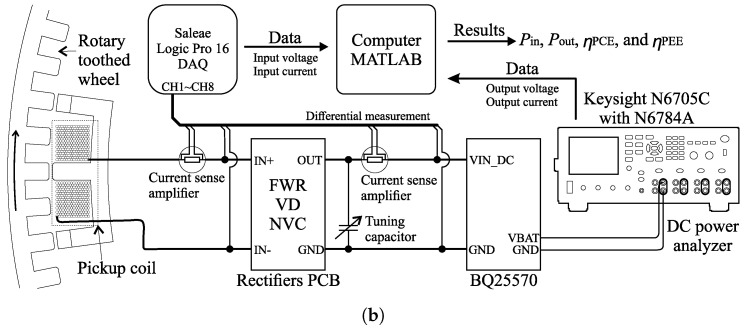
Overview of the experimental setup with the rotating shaft, the VREH transducer, the interface circuit implementations, a reference encoder, and data acquisition instruments in view of (**a**) the practical implementation and (**b**) a schematic diagram.

All experiments are conducted on a setup for rotational energy harvesting, primarily targeting industrial applications with low rotational speeds. The shaft diameter of the setup is 270 mm, and experiments are conducted under low rotational speeds in the range of 5 rpm to 20 rpm. A photo of the mechanical setup is provided in Figure 9, outlining the key components involved.

**Figure 10:**
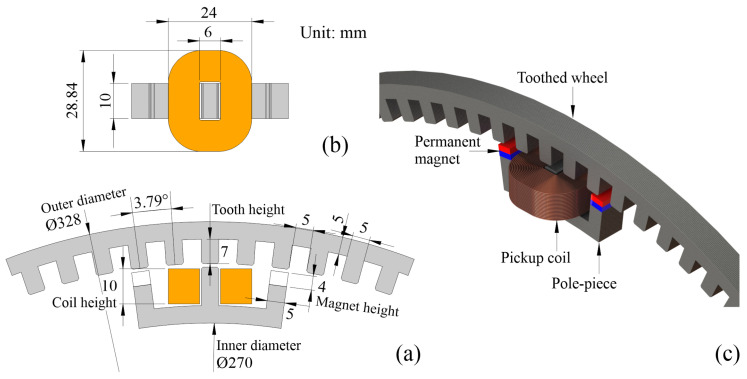
Geometric parameters in the VREH prototype (**a**) cross-sectional view, (**b**) top view (only showing the pickup unit) and (**c**) perspective.

The VREH pickup unit is mounted on a stationary POM cylinder, whereas the tooth wheel is mounted on a rotary mounting disc. The mounting disc is driven via a steel shaft by a controllable DC motor providing the relative rotary motion between pickup unit and tooth wheel with a constant air gap of 1 mm. The tooth wheel consists of 90 equally spaced teeth, and other geometrical parameters of the prototype are shown in Figure 10.

An incremental encoder provides a reference pulsed output signal under rotation and is used to monitor the rotational speeds, as well as count mechanical cycles of the toothed wheel. A flywheel is mounted on the setup to provide a stable rotational speed.

### 3.2. Measurement Setup

To characterize the system performance, input and output power of the interface circuits are measured. For the input power, the AC input current and input voltage are digitized using a Saleae Logic Pro 16 data acquisition unit (DAQ). The DAQ provides 16 channels with inbuilt 12-bit analog-to-digital converters (ADC), allowing input signals in the range of −10 V to 10 V. Prior to digitization, the input current is converted into a voltage using a current sense amplifier (uCurrent Gold). Both analog input signals are sampled at a frequency of 1 kHz.

For output power measurements, a DC power analyzer (Keysight N6705C with N6784A module) is used. The power analyzer is operated as a constant-voltage (CV) current sink with a voltage of 2.5 V, and monitors the current drain from the *VBAT* terminal of the BQ25570. The same instrument is also used to measure the intermediate power at the output of the rectifier (i.e., before the PMIC).

**Figure 11:**
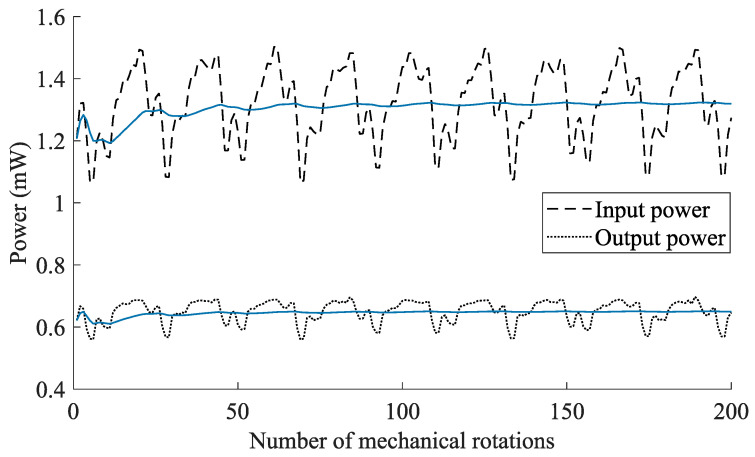
Input and output power variations caused by the mechanical setup and MPPT update rate (0.0625 Hz). The average power (blue solid lines) are obtained by taking multiple mechanical rotations into count. The example is for an interface circuit with the FWR and a tuning capacitance of 1600 μF at a rotational speed of 10 rpm.

Input power variations occur due to small air gap differences in the setup, and are further influenced by the low update rate of the MPPT circuit (0.0625 Hz) of the BQ25570. In order to minimize the effects of these variations, all measurements are conducted over a number of full rotations (200 mechanical rotations). An example for this averaging process is given in Figure 11. All acquired data is transferred to a host computer for further processing in MATLAB. As a results, $P_{\mathrm{in}}$ and $P_{\mathrm{out}}$ are obtained according to

(5) $$\begin{array}{c} P_{\mathrm{in}}=\frac{1}{T}\int_{t_{0}}^{t_{0}+T}\left[V_{\mathrm{in}}\left(t\right)I_{\mathrm{in}}\left(t\right)\right]dt\\ P_{\mathrm{out}}=V_{\mathrm{out}}\frac{1}{T}\int_{t_{0}}^{t_{0}+T}I_{\mathrm{out}}\left(t\right)dt\;, \end{array}$$

where $V_{\mathrm{in}}$, $I_{\mathrm{in}}$, $V_{\mathrm{out}}$, and $I_{\mathrm{out}}$, are the input voltage, input current, output voltage, and output current, respectively.

### 3.3. Performance Metrics

Two metrics are used to evaluate the performance of the interface circuits and their effects on the energy harvesting system performance, respectively. These metrics are the power conversion efficiency (PCE) and the power extraction efficiency (PEE).

The PCE describes the conversion efficiency of the interface circuit. It takes the efficiencies of the rectifier circuit ($\eta_{\mathrm{Rec}}$) and of the power conditioning circuit ($\eta_{\mathrm{DC}-\mathrm{DC}}$) into account, such that

(6) $$\mathrm{PCE}=\eta_{\mathrm{Rec}}\;\cdot \;\eta_{\mathrm{DC}-\mathrm{DC}}=\frac{P_{\mathrm{out}}}{P_{\mathrm{in}}}\;.$$

Herein, $P_{\mathrm{in}}$ and $P_{\mathrm{out}}$ are the input and output power levels of the interface circuit, respectively (see Figure 8b). $\eta_{\mathrm{Rec}}$ is expressed by $1-P_{\mathrm{loss}}/P_{\mathrm{in}}$, where $P_{\mathrm{loss}}$ is the power dissipated by a rectifier circuit. For diode-based rectifiers, it mainly depends on the voltage $V_{D}$ and the current $I_{D}$. For MOSFET-based rectifiers, in addition to the conduction loss due to the drain-source on-resistance $R_{\mathrm{DSon}}$, the power loss is also affected by the drain-to-source switching loss [51]. It therefore depends on the input voltage ($V_{\mathrm{in}}$), voltage frequency ($f_{e}$), threshold voltage ($V_{\mathrm{GS}(\mathrm{th})}$), load current ($I_{\mathrm{Load}}$), and the output capacitance ($C_{\mathrm{oss}}$) [29,51,52]. In this work, the switching loss can be neglected due to the low frequencies generated by the energy transducer provided in Table 3. $\eta_{\mathrm{DC}-\mathrm{DC}}$ is the conversion efficiency of the commercial PMIC (BQ25570), which is a function of input and output parameters as defined in its data sheet [53].

The PEE replaces the actual input power with the achievable power level under the current excitation, given a matched load ($P_{\max}$). As such, it takes into account how the transducer output is affected by the interface circuit. Consequently, PEE is a good measure of the energy harvesting system performance, similar to a normalized output power, with

(7) $$\mathrm{PEE}=\frac{P_{\mathrm{out}}}{P_{\max}}=\frac{P_{\mathrm{in}}}{P_{\max}}\;\cdot \;\mathrm{PCE}\;.$$

## 4. Results and Analysis

In this section, we present and analyze the results obtained from the three sets of experiments. First, in Section 4.1, the output power of the transducer under matched loads is provided. We then present and analyze results of the interface circuit performance (PCE and PEE) in Section 4.2. Finally, in Section 4.3, we evaluate effects of the different implementations on the operating conditions of the wireless RPM sensing system as an electrical load.

### 4.1. Output Power at Matched Load

Based on the experimental setup shown in Figure 8a, the maximum output power of the VREH transducer was evaluated for a matched load condition. The load resistance was selected to equal the DC resistance of the pickup coil, with $R_{\mathrm{Load}}=R_{\mathrm{Coil}}=180.5\;\Omega$. Moreover, a tuning capacitor was used to compensate for the internal inductance of the pickup coil ($L_{\mathrm{Coil}}\approx 690\;mH$, number of turns: 3000).

**Figure 12:**
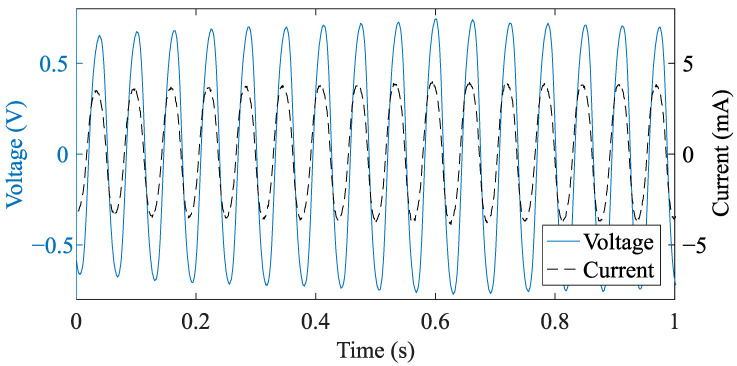
Measured waveforms of transient voltage and current, exemplified for a matched load condition, $R_{\mathrm{Load}}=180.5\;\Omega$ and $C_{\mathrm{Tuning}}=160\;\mu F$, for the VREH operating at 10 rpm.

Figure 12 illustrates an example output of the current and voltage measurement for the case of a rotational speed of approximately 10 rpm (≈1.06 rad s^−1^). According to (1), the measurement demonstrates an AC output with a frequency $f_{c}=\omega N_{t}=1.06\;\mathrm{rad}/s^{-1}\;\cdot \;90=15.15\;\mathrm{Hz}$. The measurement shows that the output current and voltage are approximately in phase, which demonstrates an effective impedance matching. Moreover, Table 3 lists the maximum output power of the VREH transducer as a function of rotational speed under matched load conditions, as well as the corresponding voltage and frequency performance parameters. These output power values (i.e., $P_{\max}$) are used as a reference for the performance evaluation of the individual interface circuit implementations.

### 4.2. PCE and PEE Evaluation

**Figure 13:**
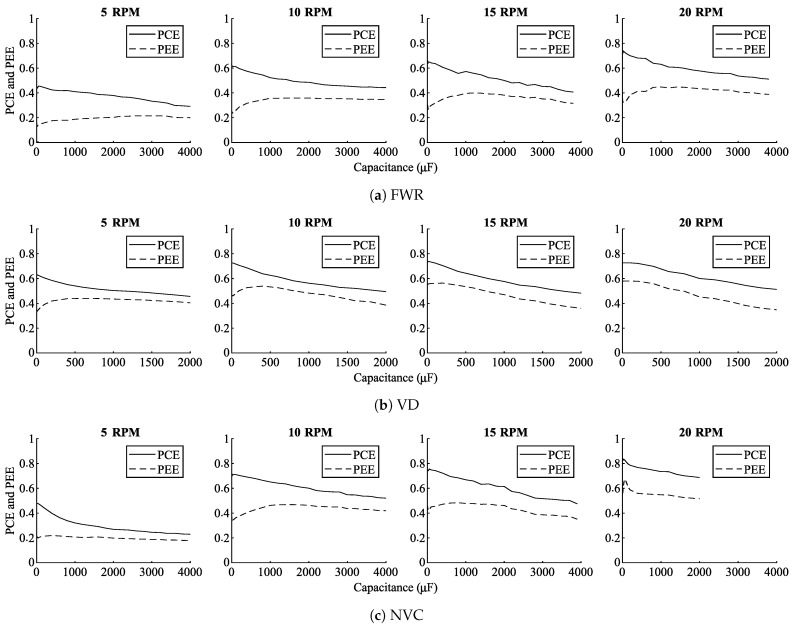
PCE and PEE development for different tuning capacitances for interface circuits based on (**a**) FWR, (**b**) VD, and (**c**) NVC, at the rotational speeds of 5 rpm, 10 rpm, 15 rpm, and 20 rpm.

Figure 13 shows the PCE and PEE estimations for the interface circuit implementations at four different rotational speeds (i.e., 5 rpm, 10 rpm, 15 rpm, 20 rpm). The results show that the circuit performance (PCE and PEE) is dependent on the used rectifier circuit and the rotational speed. It can moreover be tuned using the capacitance between the rectifier circuit and the PMIC.

It can be observed that for each condition an optimal tuning capacitance exists maximizing PCE and PEE, respectively. However, for optimization of the PCE and PEE respectively, different capacitance values are required, with optimizations of the PEE requiring a larger capacitance than optimizations of the PCE. As the PEE increases even with decreasing PCE, the results demonstrate that the tuning capacitance has an effect on the operating point of the transducer, increasing its power output. For example, at the rotational speed of 10 rpm the NVC-based system reaches its maximum PCE of 72% with a tuning capacitance of 5 μF. However, the PEE under this condition is only 36%, whereas the maximum PEE of 47% is achieved with a tuning capacitance of 1600 μF. As the PEE-metric incorporates the PCE, this shows that under this condition the increased output power of the VREH transducer outweighs the higher losses in the interface circuit.

**Figure 14:**
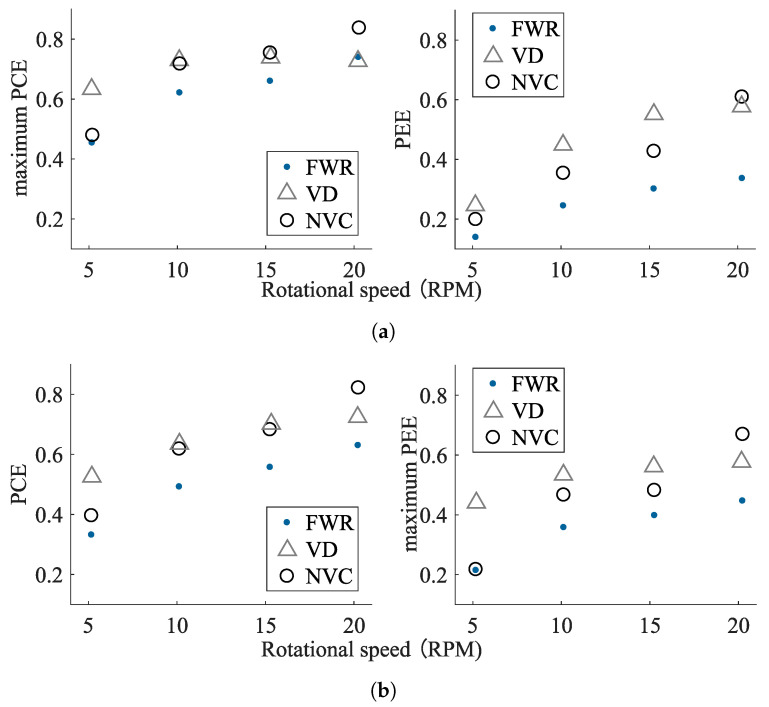
Optimized PCE and PEE values at different rotational speeds for (**a**) PCE-optimized tuning capacitance and (**b**) PEE-optimized tuning capacitance.

This becomes even more obvious when extracting the maximum PCE and PEE values for each condition, as depicted in Figure 14. Figure 14a shows PCE and PEE values for an PCE-optimized capacitance, whereas Figure 14b shows those for an PEE-optimized capacitance. It can be observed that a higher rotational speed increases PCE and PEE. This is due to the higher output voltages and currents obtained from the transducer at higher rotational speeds, which improves conversion efficiencies. Moreover, the results show that the capacitance should be tuned for a maximized PEE value, as a higher PEE is equivalent to a higher output power.

Table 4 lists the input and output conditions, as well as the individual conversion efficiencies of AC-DC and DC-DC conversion for each of the interface circuit implementations. Comparing the three rectifier circuits, the FWR demonstrates the lowest performance under all of the operating conditions. This is to be expected due to the relative low output voltages of the VREH transducer, combined with the higher forward voltage drop of the FWR circuit. As a result, the FWR provides low conversion efficiencies, particularly at low rotational speed (i.e., low input voltage), and its output levels lead to relatively low efficiencies of the PMIC as well [53]. The VD, in contrast, shows significantly higher performance values. This is a result of three effects: The VD itself provides a higher conversion efficiency due to its reduced voltage drop in comparison to the FWR; its output levels lead to a higher PMIC efficiency; and input power levels demonstrate that it extracts more power from the VREH transducer. Finally, the NVC shows a performance that is highly condition dependent. At low rotational speed (5 rpm), the NVC performs similarly to the FWR, but its performance increases rapidly with increasing rotational speed, outperforming the VD at the highest input conditions. This difference can be explained based on the characteristics of the MOSFETs in the NVC circuit. At a low rotational speed, even with the parallel connected Schottky diodes, the input voltage is far below the voltages required for the switching of the transistors, effectively converting the NVC into an FWR (see Figure 3). With increasing input voltages at higher rotational speeds the on-resistances are reduced, and thus a lower voltage drop is achieved. Although the PMIC efficiency remains below that of the VD solution, the improved rectifier efficiency, together with an increased power extraction from the VREH transducer, results in an overall increased output power.

### 4.3. End-to-End System Evaluation

For the end-to-end energy harvesting system evaluation, the VREH system is used to supply the wireless RPM sensor described in Section 2.3, and as depicted in Figure 8c. In this scenario, the interface circuit provides a regulated 1.8 V output through the PMIC’s *BUCK_OUT* terminal. An additional power conversion through the PMIC’s internal buck converter thus needs to be taken into account. The efficiency of this conversion mainly depends on the output current defined by the sensing system’s sample rate (see Table 1), resulting in efficiencies of approximately 85% to 93% for a supply voltage of 1.8 V.

Four 220 μF tantalum capacitors connected in parallel provide an energy buffer at the *VBAT* terminal. Tantalum capacitors offer a high performance in terms of volumetric efficiency, electrical reliability, and temperature range, contributing to the minimized and robust wireless sensing system. This capacitance can accumulate sufficient energy for the wireless sensor’s initialization and duty cycling operation. For the current system design, a fixed tuning capacitance is provided for each rectifier implementation. The tuning capacitance has been selected to provide a near-optimal PEE under the range of rotational speeds investigated. Based on the measurement results presented in Section 4.2, the three tuning capacitors have been selected as 2000 μF, 1000 μF, and 400 μF, for the FWR, NVC, and VD, respectively.

**Figure 15:**
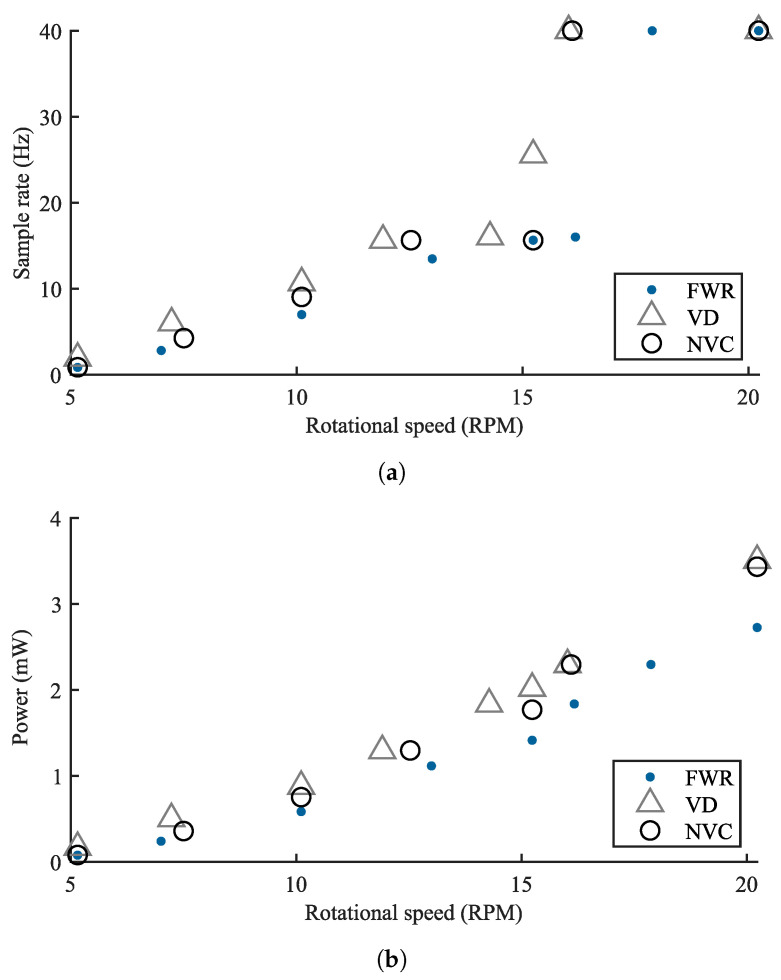
Sample rate (**a**) and power consumption (**b**) of the wireless RPM sensor operated at different rotational speeds, and supplied through the interface circuits with the three rectifiers.

Figure 15 shows the obtained output power levels and achieved sample rates of the wireless RPM sensor under different operational speeds and with the respective rectifier implementations. In accordance with the results presented in Section 4.2, the FWR implementation demonstrates the worst performance, whereas the VD implementation provides the highest performance at low rotational speeds. At 10 rpm, for example, the achievable sample rates are approximately 10 Hz, 9 Hz, and 7 Hz, for the VD, NVC, and FWR implementations, respectively.

At about 15.5 rpm, both the VD and NVC implementations reach the maximum sample rate of 40 Hz for the current system implementation. For the same sample rate, the FWR requires a rotational speed of approximately 18 rpm.

## 5. Conclusions

In this paper, three interface circuits based on passive rectifiers, namely FWR, VD, and NVC, have been compared with respect to their influences on the performance of a low-speed rotational kinetic energy harvesting system. The rectifier circuits were individually integrated with a commercial PMIC in order to experimentally evaluate the interface circuits’ conversion efficiency (PCE) and their influence on the energy harvesting system performance (PEE).

Based on a tuning capacitor, it was shown that a trade-off exists between the optimization of PCE and PEE. Although a larger tuning capacitance was shown to negatively effect the conversion efficiency of the interface circuit, the PEE and thus the total output power continues to increase up to a specific point (PEE optimum). This shows that the tuning capacitance has an influence on the operating point of the VREH transducer, which can outweigh conversion losses in the circuits.

In the comparison of the individual interface circuit implementations, significant performance differences were observed, thus indicating the importance of proper interface circuit selection based on the application conditions. At low rotational speeds, a solution based on a VD rectifier was demonstrated to outperform the other rectifiers, doubling the PEE at a rotational speed of 5 rpm. This is a result of the VD’s higher conversion efficiency, its output levels leading to a higher PMIC efficiency, as well as a higher extracted power from the VREH transducer. However, the losses in the NVC rectifier are significantly reduced at higher rotational speeds, leading to an increased output power.

The general results of the interface circuits were verified in an end-to-end energy harvesting system evaluation based on a self-powered wireless RPM sensing system. The results of this evaluation demonstrated significant differences of achievable sample rates based on the rectifier circuit selection. The highest sample rates were obtained with an VD-based interface circuit at a low rotational speed, whereas the VD and NVC performed similarly at higher rotational speeds. For the energy harvesting system implementation, a fixed tuning capacitor was selected for each interface circuit solution, leaving further performance improvement opportunities. A dynamic tuning capacitance, for example, may lead to an increased PEE, but effective implementations of this dynamic adjustment would need to be investigated. Furthermore, other solutions to improve the operating point of the the VREH transducer without negatively affecting the PCE are opportunities for further investigation.

By applying a similar optimization procedure as presented in [22], optimal dimensions of the magnet height, coil height, and tooth height can be identified to result in an VREH with highest volumetric power density for a given size. Alternatively, the size of the VREH device can be minimized for an expected output power requirement. Thus, using the optimized structure is essential to further improve the sensor performance, as well as to minimize the entire self-powered system.

In conclusion, the selection and tuning of interface circuits for low-speed electromagnetic energy harvesting systems is shown to have significant effects on the end-to-end energy harvesting system performance, demonstrating that an optimal solutions is condition-sensitive. This knowledge can be used as a design guideline for energy harvesting systems with low input voltages, and for further investigations on hybrid or adjustable solutions.

## Figures and Tables

**Table 1:** Operation modes of the wireless RPM sensor, including supported sample rate ranges and component activity level.

	Mode 1	Mode 2
Gyroscope	duty-cycled	active
BLE SoC	duty-cycled	duty-cycled
Sample rate $f_{s}$ (Hz)	(0–15.625)	(15.625–40)

**Table 2:** Specification of key components for the rectifier circuit implementations.

Component	Specification	Characteristics
C1, C2	Ceramic capacitor	16V, 100μF
D1 – D10	Schottky diode	$V_{F}=0.31V$ @ $I_{F}=0.1A$
	CTS05S40-L3F, SOD882	$I_{R}=-13\mu A$ @ $V_{R}=-10V$
		$I_{R}=-50\mu A$ @ $V_{\mathrm{BR}}=-40V$
N1, N2	N-MOSFET	$C_{\mathrm{iss}}=40pF$, $C_{\mathrm{oss}}=8.5pF$, $C_{\mathrm{rss}}=4.5pF$ @ $V_{\mathrm{DS}}=5V$
	BSS138PW-115, SOT323	$C_{\mathrm{iss}}=42pF$, $C_{\mathrm{oss}}=14pF$, $C_{\mathrm{rss}}=7pF$ @ $V_{\mathrm{DS}}=1V$
		$R_{\mathrm{DSon}}=1\Omega$, $V_{\mathrm{GS}(\mathrm{th})}=1.2V$
		$V_{\mathrm{SD}}=0.75V$ *
P1, P2	P-MOSFET	$C_{\mathrm{iss}}=1700pF$, $C_{\mathrm{oss}}=300pF$, $C_{\mathrm{rss}}=204pF$ @ $V_{\mathrm{DS}}=-5V$
	PMN27XPE-115, SOT457	$C_{\mathrm{iss}}=1800pF$, $C_{\mathrm{oss}}=500pF$, $C_{\mathrm{rss}}=305pF$ @ $V_{\mathrm{DS}}=-1V$
		$R_{\mathrm{DSon}}=27m\Omega$, $V_{\mathrm{GS}(\mathrm{th})}=-1.0V$
		$V_{\mathrm{SD}}=-0.7V$ *
* Body diode forward voltage.		

**Table 3:** Electrical performance of the VREH transducer at different rotational speeds under matched load and open circuit conditions.

	Matched Load					Open Circuit	
RPM	$P_{\max}$ (mW)	RMS Voltage (V)	Peak Voltage (V)	$R_{\mathrm{Load}}$ (Ω)	$C_{\mathrm{Tuning}}$ (μF)	Peak Voltage (V)	Frequency (Hz)
5	0.423	0.276	0.390	180.5	600	0.798	7.55
10	1.808	0.571	0.808	180.5	160	1.619	15.15
15	4.112	0.861	1.218	180.5	70	2.447	22.51
20	6.966	1.121	1.585	180.5	35	3.221	30.02

**Table 4:** Input and output conditions of the PEE-optimized interface circuits based on FWR, VD, and NVC.

		Rectifier Input			Rectifier Output			Efficiencies	
	RPM	Power (mW)	Voltage (V)	Current (mA)	Power (mW)	Voltage (V)	Current (mA)	Rectifier	PMIC
FWR	5	0.276	0.480	0.575	0.145	0.327	0.443	0.525	0.630
	10	1.310	0.822	1.593	0.811	0.619	1.310	0.619	0.801
	15	2.964	1.185	2.501	1.948	0.960	2.029	0.657	0.844
	20	4.926	1.556	3.165	3.670	1.319	2.783	0.745	0.850
VD	5	0.310	0.433	0.809	0.226	0.672	0.337	0.729	0.822
	10	1.420	0.770	1.917	1.130	1.371	0.824	0.796	0.863
	15	3.219	1.139	2.827	2.704	2.125	1.273	0.840	0.909
	20	5.477	1.414	3.952	4.697	2.617	1.795	0.858	0.912
NVC	5	0.232	0.496	0.468	0.120	0.442	0.271	0.516	0.773
	10	1.383	0.779	1.776	1.005	0.691	1.455	0.726	0.842
	15	2.906	1.214	2.393	2.281	1.149	1.985	0.785	0.872
	20	5.657	1.491	3.794	5.258	1.431	3.674	0.929	0.889

## Data Availability

Data is contained within the article.
